# A Comprehensive Study of Techniques to Optimize the Extraction of Lipids from the Autotrophic Strain of the Microalgae *Chlorella vulgaris*

**DOI:** 10.3390/life13101997

**Published:** 2023-09-30

**Authors:** Ian Foerster, Wayne Seames, Jasmine Oleksik, Alena Kubatova, Andrew Ross

**Affiliations:** 1 Chemical Engineering Department, University of North Dakota, 243 Centennial Drive Stop 7101, Grand Forks, ND 58202, USA; ian.foerster.1@und.edu; 2 Energy & Environmental Research Center, University of North Dakota, 15 N. 23rd St., Grand Forks, ND 58202, USA; joleksik@undeerc.org; 3 Chemistry Department, University of North Dakota, 151 Cornell Street Stop 9024, Grand Forks, ND 58202, USA; alena.kubatova@und.edu; 4 School of Chemical and Process Engineering, University of Leeds, 209 Clarendon Road, Leeds LS2 9JT, UK; a.b.ross@leeds.ac.uk

**Keywords:** algae, algae oil, lipids, extraction, autotrophic algae, extraction optimization

## Abstract

Microalgae represent a promising source of triglycerides and free fatty acids, synthesized in the form of lipids, for use in renewable fuels and chemicals. One challenge is the ability to efficiently recover the lipids from within the microalgae cell. Although various techniques have been studied individually, a comprehensive study of extraction techniques using consistent experimental and analytical methodology is missing. This study aims to provide this unifying comparison using the common microalgae strain *Chlorella vulgaris*. The factors that were surveyed and then optimized to achieve maximum extraction efficiency included the solvent type; mechanical pre-treatment using a ball mill at a variety of grinding speeds; microalgae-to-solvent ratio; extraction facilitated by microwave; extraction facilitated by sonication; extraction facilitated using increased temperature; and extraction facilitated by in situ transesterification to convert the lipids into esters prior to extraction. The optimum conditions determined during these studies were utilizing methanol as the solvent, with ball mill pretreatment at a grinding speed of 500 rpm, and a 1:9 microalgae to solvent ratio. When used in combination with microwave-assisted extraction at a temperature of 140 °C, approximately 24 wt% of the initial lipids were recovered. Recoveries of over 70 wt% were obtained without a microwave at extraction temperatures of over 200 °C.

## 1. Introduction

One challenge to the exploitation of microalgae as a resource for renewable fuels and chemicals is the ability to effectively recover the lipids, comprised primarily of triglycerides and free fatty acids (known collectively as FA oils), from the microalgae. Three categories of lipid extraction strategies are common in the literature: physical, chemical, and mechanical. Mechanical extraction techniques include an expeller and an oil press, both of which are commonly used with oilseed crops. However, these methods have been found to be ineffective in removing the lipids from microalgae [1]. 

Physical extraction techniques include ball milling, microwave, and sonication, which are techniques used to rupture the cell walls to allow for easier passage of lipids out of the cell. Microwave and sonication methods have been reported as effective but energy intensive methods [1]. 

Chemical extraction techniques include solvent extraction, an exhaustive extraction of microalgae via Soxhlet, and supercritical fluid extraction [2,3,4]. All of these techniques rely on a chemical solvent to extract lipids from within the algae biomass. Each technique has its strengths and weaknesses when considering factors such as toxicity, removal effectiveness, and required operating conditions. The use of Soxhlet is a common laboratory technique that yields high lipid extraction efficiencies. However, Soxhlet requires long processing times and large quantities of solvents which dilute the target extractives, requiring additional drying/separation steps [5]. These issues make Soxhlet difficult to scale to industrial processes and thus this method was not considered in this research. 

Supercritical fluid lipid extraction also benefits from high extraction efficiencies but requires very high pressures (>20 MPa) at high temperatures [4,6]. This makes supercritical fluid lipid extraction difficult to perform at a lab scale and causes increased costs to scale to industrial processes. Due to the anticipated high capital and operating costs associated with supercritical fluid lipid extraction, it was not considered in this study. 

Non-polar solvents, such as hexane, have most commonly been utilized for FA oil extraction from oilseed crops [7,8,9]. Unfortunately, when used for extraction from microalgae, non-polar solvents have limited penetration efficiency into the cell due to the strong hydrogen bonds that are formed between the membrane associated with the lipids and the proteins in the cell. To overcome this limitation, an extraction solvent using a mixture of polar and non-polar compounds has been studied. The polar compound is used to disrupt the hydrogen bonds in the cell and the non-polar compound to extract the lipids. 

Several different pure solvents and combinations of solvents have been studied at length. Some of the solvents used in different combinations include chloroform, methanol, dimethyl sulfoxide, hexane, isopropanol, ethanol, and acetonitrile [3]. 

An additional technique that might enhance solvent extraction is to react the acidic groups of the lipids with lower-order alcohols to generate esters. This technique is known as in situ transesterification [10]. Excess alcohol then acts as a solvent to extract the esters [11]. It has been postulated that in situ transesterification increases cell disruption and makes the transformed lipid more accessible for extraction. [12] For all chemical extraction methods, an efficient microalgae-to-solvent ratio is necessary if the method is to be commercially feasible. 

While the extraction of lipids from microalgae has been studied extensively at the laboratory scale [13,14,15,16], a comprehensive study of the most attractive of the various techniques using a similar and consistent experimental and analytical methodology is missing. The purpose of the research presented herein was to complete such a comprehensive study using an autotrophic version of *Chlorella vulgaris.*

## 2. Materials and Methods

### 2.1. Materials and Reagents

Two sources of autotrophic *Chlorella vulgaris* microalgae were used. The first source was a freeze-dried strain supplied by Qingdao Sunrise Trading Co., Ltd., Qingdao, China. The second source was cultured at the University of North Dakota, USA from an original strain obtained from Carolina Biological, Burlington, NC, USA (Item # 152075), and inoculated into the Bolds Basal Media (BBM) growth solution defined in Table 1 [17].

The following solvents were used in the preliminary study of potential solvents, as described in the experimental section below: chloroform, methanol, hexane, acetonitrile, ethanol, and Bligh Dyer (a mixture consisting of 66 vol% chloroform and 33 vol% methanol). Subsequent experiments were performed using methanol, ethanol, and hexane. High-purity solvents were purchased from Fisher Scientific (chloroform product number C574, acetonitrile product number A955, ethanol product number BP28184, hexane product number H2924, and methanol product number A433S20). Hydrochloric acid, purchased from Fisher Scientific (product number A142212), was utilized in the examination of the feasibility of in situ transesterification for the extraction of microalgae oil. 

Dichloromethane (DCM) purchased from Fisher Scientific (product number AC406920040) was utilized in sample preparation for thermal carbon analysis (TCA) to suspend the extraction products in solution. 

### 2.2. Experimental Setups

An International Equipment Company (IEC), HN-SII Centrifuge, was used to obtain a concentrated slurry of microalgae before freeze drying. The centrifuge was operated at 2000 rpm for 10 min with 4 × 250 mL of high-density polyethylene bottles to concentrate several liters of microalgae suspended in growth solution to less than 25 mL of slurry. 

A FreeZone Freeze Dryer (Kansas City, MO, USA) was used to dehydrate and preserve the microalgae for future use. Additionally, freeze drying acts as an initial method to crack the cell wall which aids solvent extraction. Freeze drying cracks the cell walls by first freezing the microalgae slowly to form large intracellular ice crystals before exposing the sample to a low pressure (c.a. 1 kPA) and temperature (c.a. −40 °C) causing the ice crystals to sublime to dry the microalgae [18]. Drying is an energy intensive process and wet processing may be more relevant for commercial applications. This research was performed in multiple lab spaces in different countries and due to import/export limitations, any biomass transferred between the labs had to be freeze dried. Thus, in order to ensure consistency between experimental studies, all biomass was freeze dried before extraction. 

A Retsch MP100 Planetary Ball Mill (Retsch, Haan, Germany) was used to disrupt the microalgae cell walls for selected experiments to study the ability of ball milling to facilitate more efficient solvent extraction. Planetary ball mills yield a well-mixed sample with a high degree of fineness. Additionally, planetary ball mills provide high pulverization energy which leads to shorter grinding times [16]. The revolution rate in this unit could be varied. Therefore, the rate of grinding was also explored to identify the optimum condition to disrupt the cell walls. 

A Milestone StartSYNTH Microwave Synthesis Labstation [19] (Milestone, Sorisole, Italy) was used in selected experiments to evaluate the ability of this technique to further disrupt the microalgae cell walls and to facilitate more efficient solvent extraction. 

Another wave-based method to disrupt microalgae cells is sonication. A Fisher Scientific 5.7 L Ultrasonic Bath (Fisher Scientific, Denver, CO, USA) was used in selected experiments to evaluate the ability of this technique to further disrupt the microalgae cell walls and facilitate more efficient solvent extraction.

Another method to increase the internal energy of microalgae in order to facilitate extraction is to increase the temperature of the algae–solvent solution. To explore this option, a small-scale batch reactor was built using a repurposed gas chromatography oven to allow temperature control. A turntable attached to a motor with clasps to hold each sample in place provided agitation in the reactor. The small-scale batch reactor allowed the temperature to be tested as a method to facilitate extraction without any additional cell wall disruption techniques being utilized. 

### 2.3. Experimental Methods: Microalgae Lipid Extraction

Several factors were examined to determine the effect on lipid extraction efficiency, including solvent choice, algae pretreatment through ball mill grinding speed, microalgae-to-solvent ratio, microwave-facilitated extraction, sonication-facilitated extraction, temperature-facilitated extraction, and in situ transesterification-facilitated extraction. 

A preliminary solvent screening study was completed which analyzed a wide range of solvents for lipid extraction from autotrophic microalgae. In these initial experiments, the solvents being screened were methanol, ethanol, hexane, acetonitrile, chloroform, and Bligh Dyer. Both microwave and sonication-assisted extraction were also evaluated in the screening study. All experiments were performed using a microalgae-to-solvent ratio (g:mL) of 1:10. This initial set of experiments was used to assess the extraction efficiency of each solvent and to provide the information needed to down-select to a more limited set of solvents for the experiments studying solvent extraction in combination with other techniques. 

#### 2.3.1. General Methods

**Figure 1:**
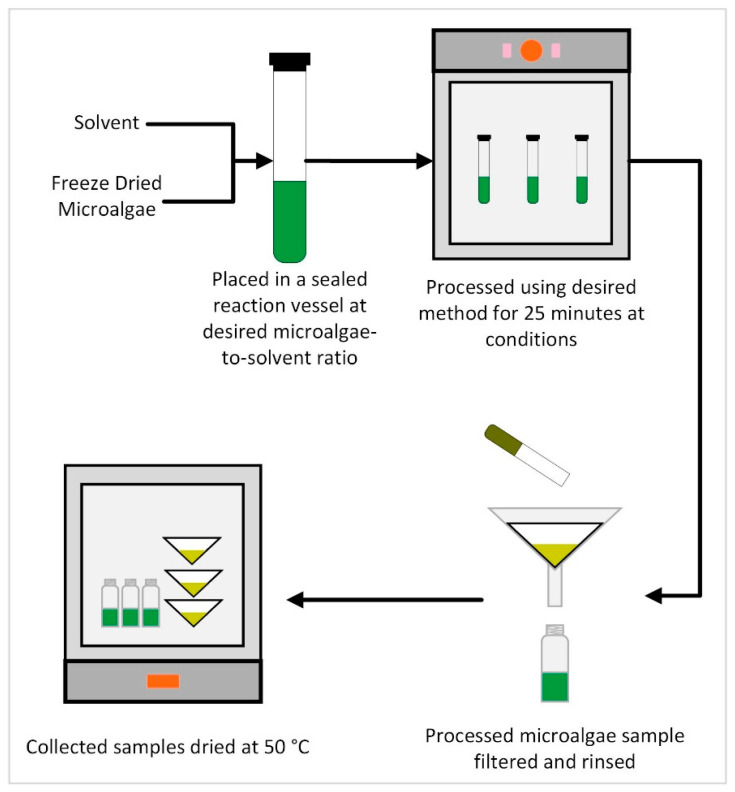
General experimental schema for the extraction method studies.

All techniques were studied using the following general method, which is summarized in Figure 1. Deviations and additions to this method are described in the subsequent Method sections. Experiments were repeated with three different solvents: methanol, ethanol, and hexane. Methanol was chosen as it was found to yield the highest extraction efficiency in the preliminary study. Ethanol was chosen to provide a similar solvent derived from a renewable feedstock, while hexane was chosen as it is the traditional solvent of choice for triacylglyceride extraction from oil seeds (e.g., soybean and canola) and thus provides a standard for comparison with other solvents and also with other FA oils generating resources (such as oil seeds). 

The efficiency of the three chosen solvents was studied and optimized by examining the following factors: ball mill grinding speed, microalgae-to-solvent ratio, microwave-facilitated extraction across several temperature profiles, sonication-facilitated extraction, temperature-facilitated extraction, and in situ transesterification-facilitated extraction.

Each optimization extraction experiment was performed in triplicate with each of the three solvents (methanol, ethanol, and hexane) using the method described below and summarized in Figure 1. Ninety-seven separate experimental conditions were chosen as summarized in Table 2. 

In general, three samples of microalgae, containing approximately 1 g of each, processed at the desired grinding speed, were weighed and inserted into the appropriate reaction vessel. Each experimental trial in the triplicate set was performed simultaneously, with each triplicate set being performed subsequently. The desired type and volume of solvent, as determined by the experimental parameters, was slowly added to the reaction vessel containing the microalgae. The reaction vessel was gently swirled by hand to help ensure even wetting and homogeneity of the microalgae/solvent mixture. The reaction vessel was then sealed and inserted into the experimental apparatus appropriate for the various factors considered (e.g., microwave, sonication, and autoclave). The microalgae and solvent were allowed to be in contact for 25 min at experimental conditions. After 25 min, the reaction vessel was removed from the experimental apparatus and emptied into a pre-weighed 12.5-cm double ring 102 filter paper. The vessel was rinsed with additional solvent to remove all residual microalgae from the vessel. The liquid was collected in a pre-weighed container. The containers of liquid were dried in a drying oven at 50 °C until a stable dry weight was achieved. The filter with the residual microalgae was also dried in a drying oven at 50 °C until any residual solvent had evaporated. The weight of the filter and the container after drying were recorded to determine the total residual microalgae and extractant.

#### 2.3.2. Grinding Study Methods

The effect of grinding as a biomass pretreatment on extraction efficiency was evaluated. Experiments were performed unground and with samples that had been ground in the ball mill at 200, 300, 400, 500, and 600 rpm. The solvent extraction procedure, described in Section 2.3.1, was then performed without any additional cell disruption techniques.

#### 2.3.3. Microalgae-to-Solvent Ratio Study Methods

The effect of the microalgae-to-solvent ratio on the extraction efficiency was completed in three studies. The first study utilized microwave-facilitated extraction at 80 °C to analyze a wide range of microalgae-to-solvent ratios following the methods described in Section 2.3.1. An operating temperature of 80 °C was chosen to allow all extraction experiments performed with methanol, ethanol, and hexane to be completed at the same temperature. Hexane experienced issues with inconsistent and unpredictable system heating when run at higher temperatures. A temperature of 80 °C was chosen to prevent this effect. The microalgae-to-solvent ratios (g: mL) which were initially studied were 1:3, 1:7, 1:11, 1:15, and 1:19. Each ratio was studied with microwave assistance and methanol, ethanol, or hexane. 

The second study utilized extraction facilitated by sonication following the methods described in Section 2.3.1 to analyze a narrow range of ratios: 1:7, 1:8, 1:9, 1:10, and 1:11 (g:mL). Each ratio was studied with sonication and methanol, ethanol, or hexane. This was followed by a third study of extraction facilitated by sonication but at a narrower range of ratios: 1:8, 1:8.5, 1:9, 1:9.5, and 1:10 (g:mL) in order to more accurately define the optimum ratio. This third study was performed only for methanol as the solvent. 

#### 2.3.4. Microwave Study Methods

The effect of microwave-facilitated extraction on efficiency was determined by performing the extraction protocol described in Section 2.3.1 with the use of microwave at several temperatures. By utilizing microwaves at various temperatures, the effect of microwaves can be directly compared to extraction efficiency facilitated by increasing temperature without microwaves. Microwave-facilitated extraction experiments were performed at 25, 50, 80, 110, and 140 °C with either methanol, ethanol, or hexane as the solvent. In this experiment, 140 °C was the upper limit due to pressure and temperature limitations in the microwave system. No results were obtained at 110 °C or 140 °C with hexane. When operating with hexane at temperatures above 80 °C, the microwave PID controller was unable to properly analyze the internal temperature of the mixture and continued to call for power to heat beyond the temperature set point. 

#### 2.3.5. Sonication Study Methods

The effect of sonication-facilitated extraction for increased efficiency was determined by performing the extraction protocol described in Section 2.3.1 with the use of sonication at 50 °C with all screening solvents, as well as the microalgae-to-solvent ratio study II, as indicated in Table 2. These results could then be compared to other cell disruption techniques for their impact on extraction efficiency. This temperature was the maximum obtainable in the sonication equipment available for this study.

#### 2.3.6. Temperature Study Methods

**Figure 2:**
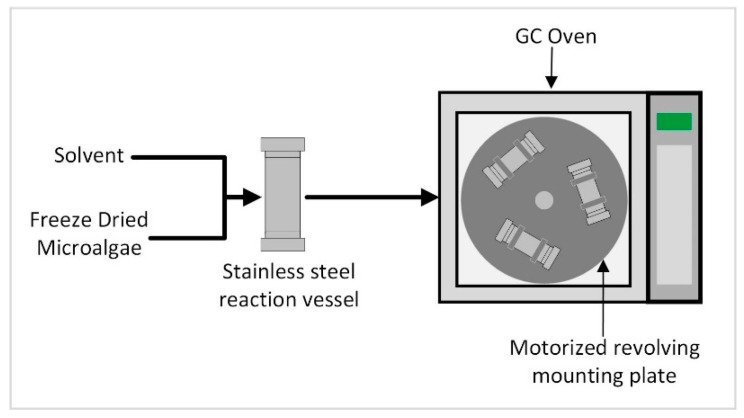
High-temperature reaction apparatus diagram.

The effect of temperature-facilitated extraction on efficiency was determined by performing the extraction protocol described in Section 2.3.1 at various temperatures. A temperature study was completed utilizing a small pressure tube reactor apparatus shown in Figure 2 and briefly described in Section 2.2. Methanol was the only solvent employed in these studies. The microalgae-to-solvent ratio was maintained at 1:10 (g/mL) and the microalgae was pretreated by grinding at 500 RPM. Approximately 0.4 g of microalgae was used in each experiment. This study was performed using the following temperatures: 25, 50, 80, 110, 140, 150, 160, 170, 180, 190, 200, 210, 220, and 230 °C. The upper limit of 230 °C was chosen to maintain sub-supercritical extraction conditions with the methanol solvent.

#### 2.3.7. In Situ Transesterification Methods

The effect of in situ transesterification-facilitated extraction on efficiency was determined by performing the extraction protocol described in Section 2.3.1 with the addition of 50 µL of hydrochloric acid into the desired type and volume of solvent, as determined by the experimental parameters. No additional alcohol was added to the solvent. The in situ transesterification experiments were performed with one feedstock, the autotrophic strain of microalgae from the University of Leeds. This technique was used in conjunction with sonication-assisted extraction at a temperature of 50 °C. 

#### 2.3.8. Analytical Methods

TCA and gravimetric analysis were used to analyze the extraction products and to determine the optimum conditions. Gravimetric analysis provided a simple non-quantitative method for trends and comparative lipid extraction efficiencies between techniques. The gravimetric results were obtained by filtering the extraction solution to separate the residual solids from the lipids-rich solvent solution. Both fractions, liquids and solids, were then dried to evaporate any remaining solvent. The solvent-free fractions were then weighed. 

TCA was chosen for the ability to quantify both esterifiable and non-esterifiable lipids, which is not possible with more common methods such as chromatographic methods [20]. Through the quantification of carbon, the TCA is more specific than the gravimetric method. TCA results were obtained from the liquid samples containing the lipids by fractioning a small portion of the fatty acids after dissolution in dichloromethane (DCM). Then, 10 µL of each sample was loaded onto a Pall Flex 2500QAT-UP tissue quartz filter (Pall Corp., East Hills, NY, USA). The sample was dried at 40 °C for 4 min to evaporate the DCM before analysis. TCA was completed utilizing a thermal optical analyzer from Sunset Laboratory Inc. (Portland, OR, USA). The TCA allows for separation of the quantified fraction based on a thermal protocol ranging from 200 to 890 °C. The lipids were assumed to elute in the 300 °C and 400 °C fractions based on the work of Lima et al. which focused on the pyrosis reactions of oils [21]. A sucrose (40 µg of loaded carbon) run was used as a daily external calibration. All samples were analyzed in duplicate. 

The TCA results from representative samples were also compared to results obtained using GC-FID following the NREL lab analytical procedure NREL/TP-5100-60958 [22]. 

## 3. Results and Discussion

The main objective of the described work was to provide a comprehensive comparison of commonly proposed techniques for the recovery of lipids from microalgae using comparable methodologies and common analytical methods. A second objective was to determine the extraction conditions that maximized the recovery of lipids from the autotrophic strain of *Chlorella vulgaris* for the techniques evaluated. In order to optimize the extraction of the lipids, several different factors were studied as shown in Table 2. For each set of experimental conditions, results for both the solid and liquid product fractions were analyzed. 

### 3.1. Solvent Study

**Figure 3:**
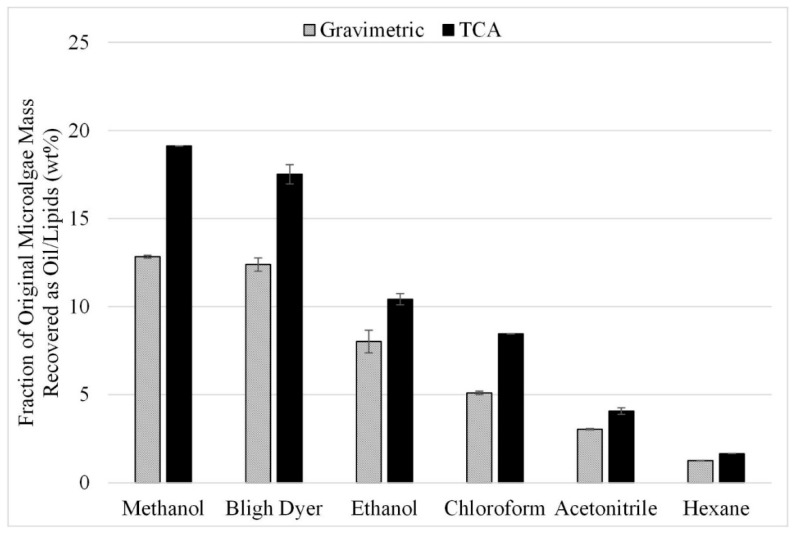
Effect of solvent choice on sonication-assisted extraction; gravimetric and TCA results. Results are presented as mean values with one standard deviation.

The first factor analyzed was solvent choice. Common solvents used in the literature as pure or mixtures include chloroform, methanol, dimethyl sulfoxide, hexane, isopropanol, ethanol, and acetonitrile [3]. As the results from these individual studies are not always consistent, a screening study was performed to identify the best solvents for a more detailed study. Gravimetric results are shown in Figure 3 from 18 experiments performed in triplicate for lipid extraction with different solvents at a temperature of 50 °C with sonication-assisted extraction. The highest gravimetric recoveries were 13 wt% for methanol and 12 wt% for the Bligh Dyer solvent (liquid recovered after solvent extraction reported as a fraction of the initial algae mass). Recovery rates for ethanol, chloroform, acetonitrile, and hexane were 8 wt%, 5 wt%, 3 wt%, and 1.2 wt%, respectively. The similar extraction efficiencies from the Bligh Dyer and methanol solvents are likely due to the methanol component in the Bligh Dyer solvent, as there is significantly lower recovery when chloroform by itself is used as the solvent (5 wt% vs. 13 wt%). 

These samples were also analyzed by TCA to provide the fraction of the original lipids recovered by each solvent (wt%). This fraction was calculated by comparing TCA results from the extracted oil sample to TCA results from the original microalgae biomass. The combined mass measured by TCA in the 300 °C and 400 °C temperature fractions for the recovered oil was divided by the combined mass in the 300 °C and 400 °C temperature fractions measured in the microalgae feedstock. Consistent with the gravimetric results, the highest overall lipid recovery as reported by TCA was 19 wt%, using methanol as the solvent.

**Figure 4:**
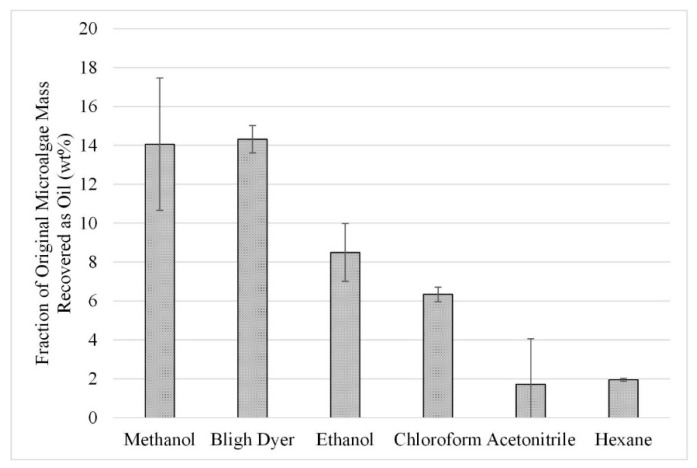
Effect of microwave-facilitated extraction on lipid extraction at a temperature of 50 °C; gravimetric results. Results are presented as mean values with one standard deviation.

The initial solvent screening experiments were repeated with microwave-assisted extraction at a temperature of 50 °C. The gravimetric results from this study can be seen in Figure 4. When comparing the results in Figure 4 to those in Figure 3, slight increases can be seen from the use of microwave-assisted extraction over those without microwave assistance. However, the uncertainty associated with methanol, ethanol, and acetonitrile does not provide statistically significant increases in extraction. Nonetheless, the trends noted for Figure 3 are also seen in Figure 4, with the best performing solvents being methanol and Bligh Dyer, with both yielding an average extraction of approximately 14 wt%.

Based on these screening experiments, methanol was selected as the primary solvent for evaluating the other facilitation methods. Ethanol was also utilized to provide a more sustainable alternative to methanol while hexane was utilized as a baseline comparison, since this is the primary solvent of choice for FA oil extraction from oilseed crops.

### 3.2. Grinding Study Results

**Figure 5:**
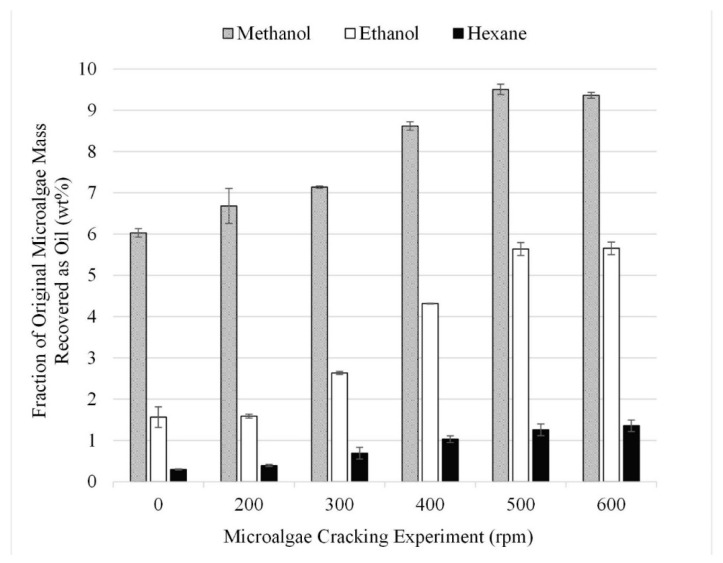
Effect of cell wall rupture due to grinding gravimetric results. Results are presented as mean values with one standard deviation.

A series of experiments were performed to analyze the impact of using grinding as a facilitation technique and to optimize ball mill speed. The gravimetric results from 18 experiments performed in triplicate for lipid extraction at different grinding conditions are summarized in Figure 5. When no grinding was employed, extraction efficiencies for methanol, ethanol, and hexane were 6, 2, and 1 wt% (dried lipids recovered after solvent extraction reported as a fraction of the initial algae mass). However, as the speed of grinding increases, so does the recovery. Therefore, it can be concluded that grinding the microalgae with a planetary ball mill increases the overall yield. 

It was determined that grinding the microalgae at 500 rpm, which resulted in 9, 5, and 1 wt% recovery for methanol, ethanol, and hexane, respectively, was the optimum pretreatment condition, as at speeds above 500 rpm no additional lipids were recovered during extraction. This conclusion is consistent with the findings from previous studies which suggest mechanical pre-treatment should be performed before performing chemical extraction [23]. 

### 3.3. Microalgae-to-Solvent Ratio Study Results

The next factor studied was the microalgae-to-solvent ratio which was studied in three phases. Finding an optimum microalgae-to-solvent ratio is a key component for scaling up to an industrial scale as handling a large quantity of solvent greatly increases the overall cost of the process. Furthermore, as the microalgae-to-solvent ratio decreases, the difficulty of separation of the solvent from the lipids increases. 

Each of the three studies was performed using microalgae pretreated at the optimum grinding speed of 500 rpm, as described in Section 2.3.2. In the first study, a wide range of microalgae-to-solvent ratios were tested based on previous literature results, with the objective of bounding the optimum ratio.

**Figure 6:**
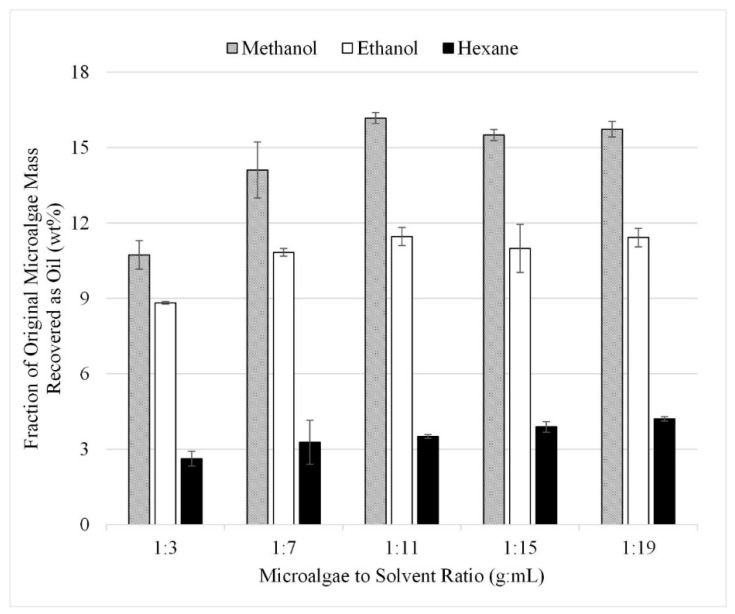
Effect of microalgae to solvent ratio with microwave gravimetric results. Results are presented as mean values with one standard deviation.

The gravimetric results from the initial 15 experiments are summarized in Figure 6. In this set, microwave-facilitated extraction at 80 °C was performed with each microalgae-to-solvent ratio. The results are presented as liquid recovered after solvent extraction reported as a fraction of the initial algae mass in wt%. The microalgae-to-solvent ratio of 1:3 produced the lowest recovery at approximately 11, 9, and 3 wt% for methanol, ethanol, and hexane, respectively. The recovery increased at microalgae-to-solvent ratios of 1:7 and 1:11 but plateaued after 1:11. It was concluded that the ideal ratio of the microalgae-to-solvent ratio is between 1:7 and 1:11 as ratios beyond 1:11 recovered no additional lipids. 

**Figure 7:**
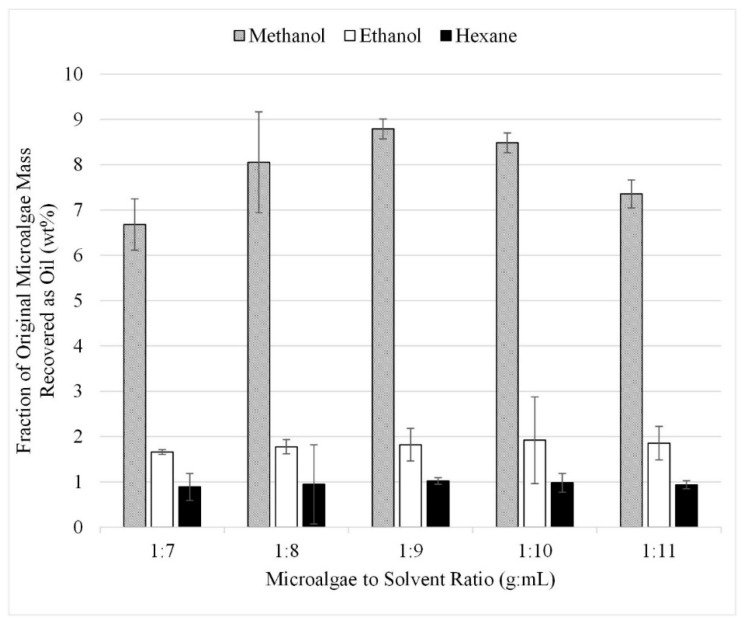
Study 2: The effect of the microalgae-to-solvent ratio with sonication on lipids extraction at ratios between 1:7 and 1:11. Results are presented as mean values with one standard deviation.

The second study analyzed a narrower range of ratios, all of which fell in between the ratios evaluated in the initial study. In this set of experiments, extraction was facilitated by sonication at 25 °C. Figure 7 presents the results as liquid recovered after solvent extraction reported as a fraction of the initial algae mass. The lower overall recoveries from the experiments in this second study compared to those from the initial microalgae-to-solvent ratio study was due to the use of sonication instead of microwave co-treatment. The recoveries measured at a ratio of 1:11 were 7.3, 1.8, and 0.9 wt% for methanol, ethanol, and hexane, respectively, whereas in the initial study using microwave-assisted extraction, the ratio of 1:11 yielded 16, 12, and 3 wt% recovery. However, the results still display the same trend and this verifies that the optimum extraction ratio falls between 1:7 and 1:11 with the highest recovery: 8.8 wt% at a microalgae-to-solvent of 1:8. From these experiments, it was determined that extraction efficiency is optimized at a microalgae-to-solvent ratio between 1:8 and 1:10 as ratios beyond 1:10 recover no additional lipids.

**Figure 8:**
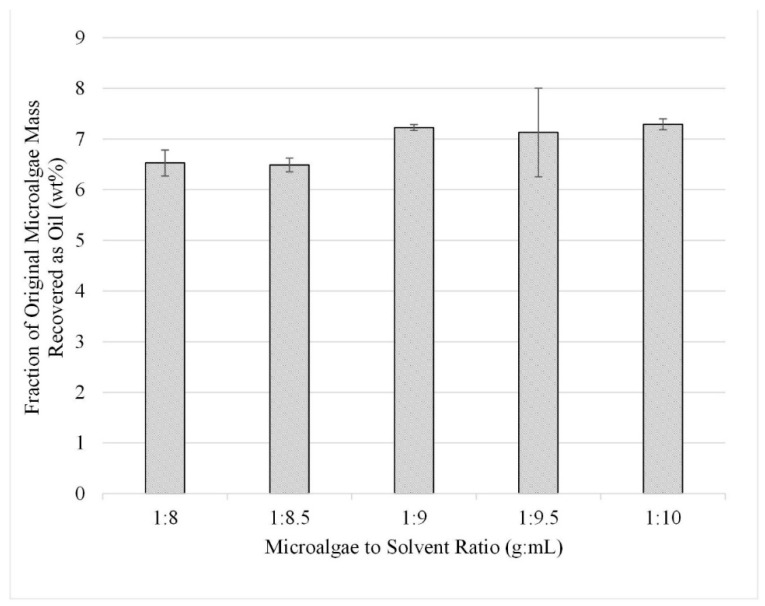
Effect of solvent-to-microalgae ratio with sonication on lipid extraction; gravimetric results. Results are presented as mean values with one standard deviation.

In the third study, five experiments were performed in triplicate for the range 1:8 through 1:10 using sonication-facilitated extraction. The results, shown in Figure 8, are displayed as liquid recovered after solvent extraction reported as a fraction of the initial algae mass (wt%). From these experiments, it was determined that the extraction efficiency of lipids from microalgae is optimized with a microalgae-to-solvent ratio of 1:9. 

The optimum solvent (methanol) and ratio (1:9) shown in Figure 8 is slightly lower than those reported in previous studies: 1:10 (g to mL) with a mixture of chloroform/methanol [24], 1:15 (g to mL) with a mixture of chloroform/methanol [25], 1:15 (g to mL) with a mixture of hexane/isopropanol [25], 1:15 (g to mL) with a mixture of hexane/isopropanol [25], and 1:10 (g to mL) just methanol [26]. Those studies reported lipid yields in the range of 2–14 wt% with respect to the quantity of lipids in the original biomass compared to the 14% found in the present work. This result is significant because the microalgae-to-solvent ratio is one of the remaining major barriers to scaling up microalgae lipid/oil extraction processes. Achieving high recoveries at lower ratios may make commercialization more feasible.

### 3.4. Microwave Study Results

Another technique analyzed was the effect of using microwaves to enhance lipid extraction. Microwaves facilitate extraction by generating heat within the cell due to friction generated by molecular movement inside the cell walls, primarily provided by the vaporization of entrained water within the cell [18]. Microwave-facilitated extraction may be a promising method to promote increased solvent extraction efficiency as it can decrease extraction time and solvent consumption [27]. 

**Figure 9:**
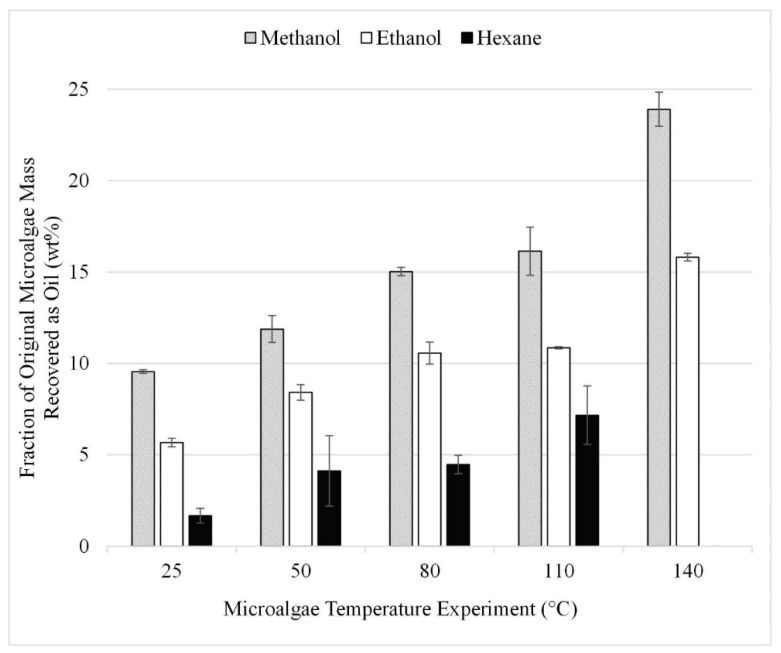
Effect of temperature during microwave-facilitated extraction on lipid extraction; gravimetric results. Results are presented as mean values with one standard deviation.

The microwave study was performed using microalgae that had been pretreated by grinding at the optimum 500 RPM and at microalgae-to-solvent ratios which fell within the near-optimum range determined in the initial microalgae-to-solvent ratio study (Section 3.3). Gravimetric results from the 15 experiments performed in triplicate for the microwave study are summarized in Figure 9. As microwave temperature is increased, lipid recovery is increased with a maximum of 24 wt% achieved at the highest temperature tested, 140 °C. The comparable result for ethanol was 15 wt%. Due to microwave temperature limitations, the optimum operating conditions were not bounded. 

A review article by Menegazzo and Fonseca [1] highlights a variety of methods studied to enhance lipid recovery from microalgae and indicates that microwave-facilitated extraction yields the highest recovery, which is consistent with the findings of the current study. Previous studies report similar results for microwave-facilitated extraction. One such study yielded 17% lipids with microwave-facilitated extraction of *Chlorella vulgaris* at 100 °C with a reaction time of 5 min [1] while a second study reported 11% lipid recovery using microwave-facilitated extraction of *Chlorella vulgaris*. The discussed yields from the literature are assumed to be the percent recovery of initial microalgae feedstock [28].

However, we observed that the combined effect of increasing extraction temperature with the presence of microwaves had a greater effect on extraction than the effect of microwaves alone. Additionally, to the best of our knowledge, the use of temperature to facilitate extraction without other extraction methods has not been reported. This observation led to further testing via temperature facilitation to compare the separable effects of microwave and temperature, as described in Section 3.6, below. 

### 3.5. Sonication Study Results

The effect of sonication on the enhancement of lipid extraction was also explored. Sonication facilitates extraction by providing ultrasonic waves to cause disruption to the cell wall through rapid compression and decompression cycles. The cycling of ultrasonic waves produces cavitation which will instigate changes in the cell wall [18]. We postulated that sonication-facilitated extraction could aid in solvent extraction through a technique that is less energy intensive than microwave-facilitated extraction. 

The sonication study was performed simultaneously with the initial microalgae-to- solvent ratio study described in Section 3.3. The gravimetric results from the 20 experiments performed in triplicate are shown in Figure 7. A comparison of the recoveries in Figure 7 to those in Figure 5 suggests that sonication at the energy levels and temperatures used in this study did not improve recovery over just solvent extraction after grinding. With all other conditions identical except for with or without sonication for both sets of experiments, recoveries were approximately the same. For example, at a microalgae-to-solvent ratio of 1:10, this corresponds to 9 and 6 wt% of the initial microalgae mass, for methanol and ethanol, respectively. Sonication does appear to improve the recovery with hexane as sonication yields approximately a 3 wt% increase. 

Figure 7 and Figure 9 can be used to compare the two techniques of cell disruption: sonication and microwave. In both figures, data are shown for lipids extracted from microalgae using the microalgae-to-solvent ratio of 1:10 at 25 °C with methanol, ethanol, and hexane. The extraction result is approximately 4% higher for microwave extraction than sonication. 

Previous studies have yielded similar results to this current study. All of the experiments in the literature completed with *Chlorella vulgaris* and sonication were performed at a microalgae-to-solvent ratio of 1:10 or 1:15 (g:mL). Maximum extraction yields from sonication-facilitated extraction with *Chlorella vulgaris* range from 7% to 14% [3,28,29], with previous work performed with similar conditions to the experimentation performed in this study. The recovered fraction is assumed to be the fraction extracted from the initial biomass and is therefore directly comparable to the results of the gravimetric analysis. Extraction with sonication was determined to be less efficient in comparison to microwave-facilitated extraction and temperature-facilitated extraction. 

### 3.6. Temperature Study Results

The effect of temperature on the enhancement of lipid extraction was also studied. The temperature study was performed using microalgae pretreated at the optimized grinding conditions and at a microalgae-to-solvent ratio in the near optimum range as determined in the initial microalgae-to-solvent ratio study. We postulated that increasing the extraction temperature within the reactor may provide the energy necessary to facilitate solvent extraction of the lipids comparable to the energy provided by microwave or sonication; eliminating the need for highly energy-intensive cell disruption techniques. If true, this may improve the scale-up potential of the extraction technique by reducing both capital and operating costs. 

**Figure 10:**
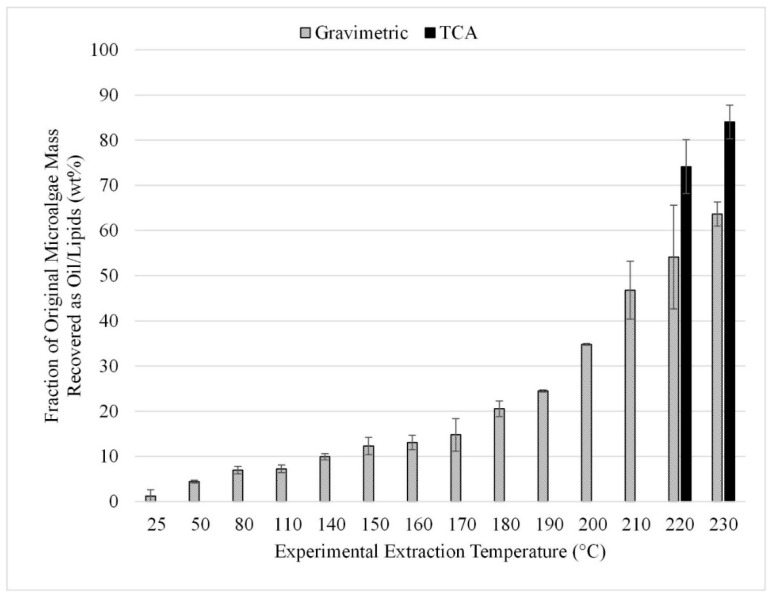
Effect of extraction temperature with a methanol solvent; gravimetric and TCA results. Results are presented as mean values with one standard deviation.

The temperature study was performed using temperatures from 25 to 230 °C. The upper limit of 230 °C was chosen to maintain sub-supercritical extraction conditions with the methanol solvent. The gravimetric results from the 12 experiments performed in triplicate are summarized in Figure 10. TCA results for the 220 and 230 °C trials can also be seen in Figure 10. These results can be compared to those shown in Figure 9 which provide the results from microwave-facilitated extraction. Comparing the gravimetric results at 140 °C for methanol, it was found that microwave-facilitated extraction was more efficient at 24 wt% compared to the approximately 10 wt% achieved at the same temperature but without the application of microwaves. 

To achieve comparable recovery without microwaves, an extraction temperature of 190 °C or greater is required (Figure 10). Thus, we can conclude that when extraction is performed within the temperature operating range of the microwave system, using the microwave provides increased lipid extraction with methanol solvent compared to extraction at the same temperature without the use of microwaves. However, at temperatures over 200 °C, the recovery increases substantially with over 70 wt% recovered (TCA method) even without microwave assistance. 

As with the microwave-assisted extraction results, the optimum temperature was not bounded during the study, as increasing temperature provided increased lipid recovery throughout the entire temperature range tested. The use of supercritical fluids like CO_2_ for lipid extraction from microalgae is commonly reported in the literature with extraction yields ranging from 25 to 90%. However, the use of supercritical pure methanol does not appear to be well documented [6]. The tradeoff for increasing into the supercritical regime is between increased recovery efficiencies versus higher capital and operating costs for equipment that can handle supercritical conditions.

### 3.7. In Situ Transesterification Study

**Figure 11:**
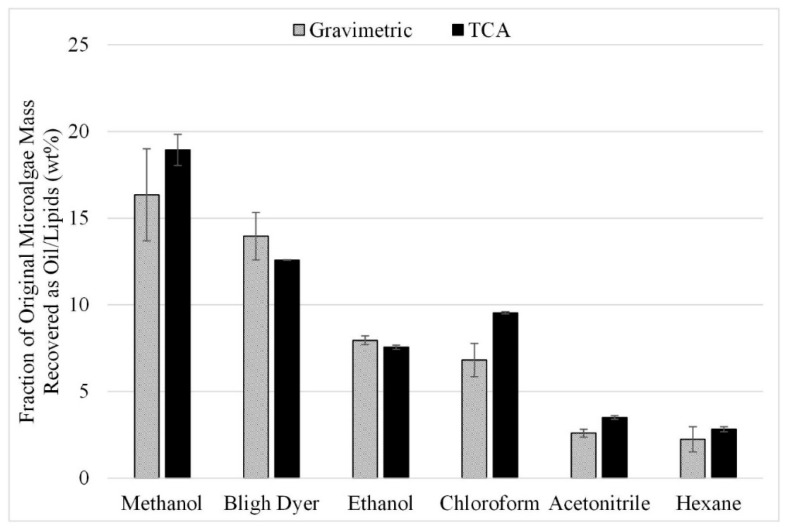
Effect of In Situ Transesterification on Lipid Extraction with sonication; gravimetric and TCA results. Results are presented as mean values with one standard deviation.

In another set of experiments, the effect of in situ transesterification for the enhancement of lipid extraction was studied. The in situ transesterification study was completed in two parts. First, six different solvents were tested since the best solvent for extraction of the esters might not be the same as that identified for the acid form of the lipids. The extraction efficiency of these solvents with sonication was determined at the following conditions: 500 rpm grinding speed, 1:10 microalgae-to-solvent ratio, and 50 °C temperature. The gravimetric and TCA results from the six experiments performed in triplicate for the in situ transesterification study are shown in Figure 11. 

The results are consistent with those from lipid acid solvent extraction (Figure 3); methanol and Bligh dyer yielded the highest recoveries with or without transesterification. Recoveries after transesterification are significantly higher than those shown in Figure 3 for extraction without transesterification under comparable conditions. However, these recoveries were not an improvement over the recovery experienced from microwave-assisted extraction. The esters recovered during the in situ transesterification experiments with methanol and ethanol were approximately 16 and 14 wt% compared to lipids recoveries of 24 and 16 wt% for 140 ° C microwave-facilitated extraction with methanol and ethanol (Figure 9). 

It should be noted that the in situ transesterification-facilitated extraction in the present study yielded lower recoveries than was reported by Chamola et al. Their results, based on utilizing a reaction temperature of 50 °C, 60-min reaction time, a 1:10 (g:mL) microalgae-to-solvent ratio, and a 1:2.8 (g:mL) microalgae to catalyst ratio, indicate that a 90% overall recovery of esters can be achieved for an acid-catalyzed reaction and 87% overall recovery for a base-catalyzed in situ transesterification reaction of free fatty acids to esters coupled with solvent extraction [30]. However, in a study by Deshmukh et al. that utilized a reaction temperature of 58 °C, a 90-min reaction time, a 1:15 (g:mL) microalgae-to-solvent ratio, and a 1:1.5 (g:mL) microalgae to catalyst ratio, a recovery of only 5.4% from the initial biomass [3] was reported, which is lower than the present study. Both of the previous studies utilized a longer reaction time and higher concentration of acid in the experimental mixture in comparison to the current study. It can also be noted that studies often have inconsistent reporting of overall recovery. When reporting results as a percentage of recovered esterifiable lipids, the results can be very high (>90% for *chlorella* sp.), as seen with Chamola et al., but the weight percent of esterifiable lipids within the algae biomass may be quite low (5–12 wt % for *chlorella* sp.), as was seen in Deshmukh et al. [12]

The impact of temperature on transesterification-facilitated extraction was not pursued since the recovery of the original lipids at high temperatures (over 200 °C) allows very high recoveries with methanol. Since the acid forms are easier to separate out of methanol than the ester forms, recoveries from transesterification-facilitated extraction would need to be substantially higher than lipid extraction for this method to be preferred.

## 4. Conclusions

This study provides a comprehensive comparison of various previously documented techniques for the extraction of lipids from microalgae and provides near-optimum conditions for each technique as well as combinations of techniques. The techniques studied included solvent choice, mechanical pre-treatment, microalgae-to-solvent ratios, microwave-facilitated extraction, sonication-facilitated extraction, temperature-facilitated extraction, and in situ transesterification-facilitated extraction. The best conditions identified during the study were determined to be utilizing methanol as the solvent, with a grinding speed of 500 rpm and a 1:9 microalgae-to-solvent ratio. It is unclear from the study as to whether the microwave or in situ transesterification methods are worth the additional costs associated with them compared to temperature-facilitated extraction alone.

Concerning the extraction temperature, for both microwave-facilitated and non-microwave-facilitated extraction, the maximum temperature tested yielded the best results. Higher temperature microwave-assisted extraction was not possible in the available system but should be explored in future work. Similarly, temperature-facilitated extraction was limited to sub-supercritical conditions in this study. Supercritical extraction at higher temperatures should also be explored in future work. In both cases, a techno-economic study would be needed to determine the optimum condition since both capital equipment and operating costs rise greatly at higher temperature conditions.

The results presented utilized TCA and gravimetric analytical methods. For comparison, results from the highest recovery case which used grinding at 500 rpm, a microalgae-to-solvent ratio of 1:10, and a temperature of 230 °C were analyzed by GC-FID in addition to the gravimetric and TCA methods. It was observed that the TCA reported the highest overall lipid recovery at 84 wt% with the GC-FID reporting the lowest recovery at 36 wt%. This large disparity is attributed to the inability of the GC-FID method to quantify non-esterifiable lipids. This agrees with previous work, which found approximately half of all lipids within *Chlorella vulgaris* algae samples to be non-esterifiable, using similar solvents [31]. The gravimetric results from this same sample yield a lipid recovery of 64 wt%, which lies between the GC-FID and TCA recoveries. Gravimetric results, although not identical to the TCA, provided adequate insight into data trends and were considered as a useful method to quickly evaluate lipid extraction experiments. 

Due to shipping regulations, all microalgae samples utilized in these experiments were freeze dried prior to use. Drying the microalgae after harvesting may represent a major operating expense. Furthermore, freeze drying partially disrupts the cells, thus acting as another pretreatment step in the process. It is recommended that the optimum techniques and conditions are verified and reoptimized for wet processed microalgae samples.

## Figures and Tables

**Table 1:** BBM Growth Media Composition.

Compound (Source)	Quantity
Sodium Nitrate (Fisher Scientific, Waltham MA, USA, 7631-99-4)	25 g
Calcium Chloride (Fisher Scientific, C70-500)	2.5 g
Magnesium Sulfate Heptahydrate (Fisher Scientific, 10034-99-8)	7.5 g
Dipotassium Hydrogen Phosphate (Sigma Aldrich, St. Louis, MO, USA, P3786-100G)	7.5 g
Potassium Dihydrogen Phosphate (Fisher Scientific, 7778-77-0)	17.5 g
Sodium Chloride (Fisher Scientific, 7647-14-5)	2.5 g
Zinc Sulfate Heptahydrate (Fisher Scientific, AC205982500)	8.8 mg
Manganese Chloride Tetrahydrate (Fisher Scientific, M87-100)	1.4 mg
Molybdenum Trioxide (Fisher Scientific, ICN15254880)	0.71 mg
Copper Sulfate Pentahydrate (Fisher Scientific, 60-004-59)	1.6 mg
Cobalt Nitrate Hexahydrate (Fisher Scientific, AC213091000)	0.49 mg
Ethylenediaminetetraacetic Acid (Sigma Aldrich, ED2SSS-50G)	9.3 mg
Acidified Iron Stock Solution (Fisher Scientific, 7782-63-0)	3 mg
Boric Acid (Fisher Scientific, A74-500)	5.7 mg
Distilled Water	1 L

**Table 2:** A Summary of the Experiments Performed in These Studies.

Experimental Set 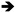	Grinding Study	Solvent Screening Study	Microalgae to Solvent Ratio Study I	Microalgae to Solvent Ratio Study II	Microalgae to Solvent Ratio Study III	In Situ Transesterification Study	Microwave Study	Temperature Study
Number of Experiments	15	36	15	15	5	4	15	12
Microalgae Type ^1^	UoL	UoL, UND	UoL	UoL	UoL	UoL	UoL	UoL
Solvent ^2^	M, E, H	M, E, H, C, AC, BD	M, E, H	M, E, H	M	M, E, H, C, AC, BD	M, E, H	M
Mill Grinding Speed (RPM)	200, 300, 400, 500, 600	250	500	500	500	500	500	500
Microalgae-to-Solvent Ratio (g_biomass_/mL)	1:10	1:10	1:3, 1:7, 1:10, 1:11, 1:15, 1:19	1:7, 1:8, 1:9, 1:10, 1:11	1:8, 1:8.5, 1:9,1: 9.5, 1:10	1:10	1:10	1:10
Temperature (°C)	25	50	80	25	25	50	25, 50, 80, 110 140	25, 50, 80, 110, 140, 150, 160, 170, 180, 190, 200, 210, 20, 230
Microwave-Assisted ^3^	-	+	+	-	-	-	+	-
Temperature-Assisted ^3^	-	+	-	-	-	+	-	+
Sonicator-Assisted ^3^	-	+	-	+	+	+	-	-
Transesterification (HCl Addition) ^3^	-	-	-	-	-	+	-	-

^1^ UoL = Microalgae obtained at University of Leeds, UK; UND = Microalgae grown at University of North Dakota, USA; ^2^ M = methanol; E = ethanol; H = hexane; C = chloroform; AC = acetonitrile; BD = Bligh Dyer. ^3^ + = Method employed in listed experiments, - = Method not employed in listed experiments.

## Data Availability

The data presented in this study are available in the following two sources: (1) Foerster, I.M., The production of bio-based chemicals and materials from renewable sources, Doctoral Dissertation, University of North Dakota, 2021 and (2) Kreft, J.L., The study of the easibility of growth, extraction, and industrial scale up processing of microalgae lipids, Masters Thesis, University of North Dakota, 2020.
